# Selectivity in associative learning: a cognitive stage framework for blocking and cue competition phenomena

**DOI:** 10.3389/fpsyg.2014.01305

**Published:** 2014-11-12

**Authors:** Yannick Boddez, Kim Haesen, Frank Baeyens, Tom Beckers

**Affiliations:** ^1^Centre for the Psychology of Learning and Experimental Psychopathology, Faculty of Psychology and Educational Sciences, KU Leuven – University of Leuven, LeuvenBelgium; ^2^University of Amsterdam, AmsterdamNetherlands

**Keywords:** learning theory, associative learning, conditioning, cue competition, stimulus competition, blocking, prediction error

## Abstract

Blocking is the most important phenomenon in the history of associative learning theory: for over 40 years, blocking has inspired a whole generation of learning models. Blocking is part of a family of effects that are typically termed “cue competition” effects. Common amongst all cue competition effects is that a cue-outcome relation is poorly learned or poorly expressed because the cue is trained in the presence of an alternative predictor or cause of the outcome. We provide an overview of the cognitive processes involved in cue competition effects in humans and propose a stage framework that brings these processes together. The framework contends that the behavioral display of cue competition is cognitively construed following three stages that include (1) an encoding stage, (2) a retention stage, and (3) a performance stage. We argue that the stage framework supports a comprehensive understanding of cue competition effects.

## INTRODUCTION

Associative learning refers to a change in behavior due to regularities in stimulus presentation (De Houwer et al., 2013). Interestingly, this definition remains silent on the psychological process that underlies learning. Arguably, that process is mostly informed by stimulus arrangements that do not produce the expected change in behavior. The blocking procedure is the most famous example of these stimulus arrangements. In this learning procedure, a cue of interest is trained together with another cue that already predicts the occurrence of the outcome. That is, pairings of two cues with the outcome are preceded by pairings of only one of both cues with the outcome (i.e., A+ followed by AX+ training). kamin (unpublished, p. 5) pioneered this procedure, by presenting rats with pairings of a white noise stimulus with shock (A+) followed by pairings of a compound of the white noise stimulus and a new light stimulus with shock (AX+), and made the famous observation that “prior conditioning to an element might block conditioning to a new, superimposed element.” This result has since been replicated many times, supporting the idea that preparatory responding to a redundant cue remains low, despite its being paired with the outcome. The blocking effect has been demonstrated with diverse learning procedures — for example using appetitive and aversive learning protocols (Kamin, unpublished; Jennings and Kirkpatrick, 2006), taste-aversion protocols (Willner, 1978), spatial learning (Rodrigo et al., 1997), and human causal learning (Dickinson et al., 1984). In addition, there is evidence of blocking in a variety of species including snails (e.g., Acebes et al., 2009; Prados et al., 2013), honeybees (e.g., Blaser et al., 2004), goldfish (e.g., Tennant and Bitterman, 1975), rats (e.g., kamin, unpublished), and humans (Dickinson et al., 1984).

Blocking is part of a family of effects that are typically termed “cue competition” effects. Common amongst all cue competition effects is that a cue-outcome relation is poorly learned or poorly expressed because the cue is trained in the presence of an alternative predictor or cause of the outcome. For example, Pavlov (1927) reported that mere compound training of two stimuli (i.e., AX+) resulted in lower preparatory responding to each individual cue relative to if the stimuli were trained elementally (i.e., A+/X+; i.e., overshadowing). Importantly, in both blocking and overshadowing, the cue of interest is trained in compound with a competing cue, but in blocking, the competing cue is previously paired with the outcome, whereas in overshadowing, the competing cue is initially neutral. Consequently, overshadowing is often used as a control for blocking because this control isolates the effect of prior training of the competing stimulus (see Arcediano et al., 2001, pp. 355–356, for an excellent discussion of the blocking control condition). Other demonstrations of cue competition effects include probabilistic cue effects and relative validity, in which the cue of interest (X) is reinforced in the presence of one cue (A) and not reinforced in the presence of another cue (B) (i.e., AX+/BX-; Wagner et al., 1968; Baker et al., 1993; Krushke and Johansen, 1999). Interestingly, the order of training in a normal blocking procedure can also be reversed (i.e., AX+ followed by A+ training; e.g., Shanks, 1985), resulting in a form of retrospective cue competition, called backward blocking.

For over 40 years, blocking has inspired a whole generation of influential learning models (e.g., Rescorla and Wagner, 1972; Mackintosh, 1975; Pearce and Hall, 1980; Wagner, 1981; Miller and Matzel, 1988; Mitchell et al., 2009). Dominant theories of associative learning typically rely on low-level link formations (i.e., associations) to implement learning at the algorithmic level, that is the level of psychological processes (Marr, 1982). Consequently, such models are termed association formation models (AFMs), as learning is characterized by the trial-by-trial formation of unqualified links that transmit activation from one mental representation to another, very much like a piece of copper wire conducts electricity. Some AFMs moreover invoke additional cognitive processes or mental states that presumably facilitate and/or enable link formation, such as attention and expectancy discrepancy (e.g., Rescorla and Wagner, 1972; Mackintosh, 1975; Pearce and Hall, 1980), amongst others. The psychological interpretation given to the influential Rescorla–Wagner model (Rescorla and Wagner, 1972), for example, states that the change in associative strength is determined by the amount of expectancy discrepancy (“prediction error”): In a blocking procedure, the preceding A+ training renders the outcome to be expected on AX+ trials, and therefore, an associative link between cue X and the outcome presumably cannot form. In contrast to AFMs, inferential learning theorists have challenged the standard presupposition that learning is based on link formation. They argue that link formation is a superfluous concept and as an alternative they argue that all associative learning effects, including cue competition phenomena, can be explained by the interplay between memory for propositions and higher-order reasoning (e.g., De Houwer, 2009; Mitchell et al., 2009). In a blocking procedure, for example, subjects supposedly infer that the blocked cue is unlikely to be (causally) related to the outcome, because the relation between the blocked cue and the outcome disappears if one controls for the relation between the blocking cue and the outcome. Accordingly, whenever the non-efficacy of the blocked cue can be validly inferred (see further in this paper), subjects should display blocking. The sometimes-intense debate between AFMs and inferential learning theories and their rivaling accounts of cue competition have been reviewed elsewhere (e.g., De Houwer and Beckers, 2002; De Houwer et al., 2005; Pineño and Miller, 2007; Shanks, 2010; McLaren et al., 2014) and are beyond the scope of the present paper.

In the present paper, we will present a cognitive stage framework for understanding blocking and related cue competition effects. Our aim is to discuss and organize the cognitive processes that play an important role in cue competition effects. In line with the suggestions of Meiser (2011), we will focus on cognitive processes that can be applied in a variety of research paradigms. AFMs were specifically developed to account for how regularities in stimulus presentation result in changes in behavior. But paradigm-specific theoretical models may fail to identify processes that are operating in other research paradigms. This hinders the development of more integrative frameworks with a broader range of applicability that go beyond the paradigm at hand (Meiser, 2011). Thus, where possible, we will invoke cognitive processes that are known to facilitate behavioral adaptation in research paradigms other than the associative learning paradigm. More precisely, we will consider processes like perception, attention, working memory, memory, higher-order reasoning and inhibition.

In the first section of this paper (“Cognitive processes in cue competition”), a number of cognitive processes and their involvement in cue competition are described. While doing so, we will additionally try to bridge the gap between construct formulations in mainstream cognitive psychology (see Neisser, 1967 and Lindsay and Norman, 1972 for texts influential in defining mainstream cognitive psychology) and construct formulations in the field of associative learning. In the second section of this paper (“A cognitive stage framework for cue competition”), a stage framework is presented and is used to integrate the cognitive processes that are involved in cue competition. Existing theories typically invoke just one or two cognitive processes, which may have led to ignoring or underemphasizing interplay. Here, we attempt to correct this oversight by providing a framework that integrates processes. Following the presentation of our framework, we will demonstrate how this framework can inspire new empirical research in the General Discussion. Among the family of cue competition effects, we will mainly focus on the blocking effect, because of its special status in associative learning theory.

## SECTION 1: COGNITIVE PROCESSES IN CUE COMPETITION

For starters, we will discuss several cognitive processes that are involved in cue competition phenomena. These cognitive processes will be discussed according to the following main headings: perception, attention, working memory, higher-order reasoning, memory, and inhibition. As said, we will cite studies and mechanisms from both the association formation tradition and the mainstream cognitive psychology tradition, and try to connect both traditions where possible. The cognitive processes discussed in this section of the paper will serve as building blocks to engineer the stage framework presented in the second section of the paper.

### PERCEPTION

A failure to perceptually process a cue represents an obvious pathway to later behavioral display of cue competition: The subject must detect the stimulus during the learning episode in order to come to respond in its presence (see Pearce and Bouton, 2001, for a similar argument). The hypothesis that lack of perception might lead to cue competition effects is consistent with most views in cognitive psychology, which assume that the processing capacity for perception is limited. In line with this, neuroscientists have argued that stimuli compete for neural representation because of the limited processing resources of the brain (e.g., Desimone and Duncan, 1995; Pessoa et al., 2002; Bishop, 2008). Unfortunately, there are, to the best of our knowledge, virtually no studies that directly relate this issue to the development of cue competition effects. This lack of data arises primarily from the fact that in “most Pavlovian paradigms, the conditions are so simple and impoverished that they probably do not come close to overtaxing an animal’s capacity to process such information” (Blaisdell, 2003, p. 148). So, although in most studies there is no reason to expect a deficit in perception in a blocking condition (as compared to an overshadowing control condition), it could play a role in (more naturalistic) situations involving many stimuli. It is of note that cue competition may not only involve cases of failing to perceive a cue during the learning episode, but a failure to perceive it at test as well (due to a change in perceptual context; Pearce, 1987, 1994). Further discussion of this latter possibility is reserved for the section on memory retrieval.

### ATTENTION

Although perception needs more investigation in cue competition research, the construct of attention, defined as the prioritized processing of information at the cost of other information (Allport, 1989), has long been the focus of theoretical consideration in the cognitive and associative learning fields.

According to Mackintosh (1975), the amount of attention devoted to cues is modulated by the relative predictiveness of those cues: Relative to the other cues present during training, more attention is paid to a cue on the next trial if it was a better predictor of the outcome on the previous trial(s), whereas less attention is paid to a cue on the next trial if it was a poorer predictor of the outcome. The Pearce–Hall model (Pearce and Hall, 1980) also focuses on the role of attention in learning, but in contrast to Mackintosh (1975), it states that more attention will be allocated to cues that are followed by a surprising outcome, relative to cues that are followed by an unsurprising outcome. Both of these models account for cue competition effects, including the blocking effect, by assuming that the amount of attention allocated to a cue determines learning and therefore responding. In a more traditional cognitive framework, such allocation of attention might be thought of as attentional regulation. Top–down attention is regulated by task-demands and goal-directed intent of the observer (e.g., Desimone and Duncan, 1995; Pessoa et al., 2002; Bishop, 2008). It has been proposed that organisms use top-down control as a way to direct their attention to “whatever information is relevant to current behavior” so as to allow the organism to adapt its behavior to the current environment (Desimone and Duncan, 1995; p. 199). Attentional disengagement from redundant information is considered an essential part of such top–down regulation (e.g., Hopfinger et al., 2000). In a blocking procedure, this might be evident in attentional disengagement from the blocked cue, which is redundant to solving the task, during training. Top–down attention differs from bottom–up attention, which is determined by properties of the stimuli. Interestingly, a recent study shows that the predictive value of a stimulus might automatically direct attention to that stimulus (Le Pelley et al., 2013). This suggests a potentially important role for bottom–up attention during blocking training as well.

Empirical evidence for the role of attention in cue competition procedures includes demonstrations that new learning about a blocked cue is retarded: Blocking treatment interferes with subsequent learning, even when an outcome different from the one during blocking training is used. This interference effect is presumably due to a decrease in attention paid to the blocked cue, caused by the preceding blocking treatment (e.g., Mackintosh and Turner, 1971; Le Pelley et al., 2007). As noted by Beesley and Le Pelley (2011), such data are, however, preliminary because they only implicate attention in cue competition under the aforementioned assumption that the amount of attention paid to a cue modulates how rapidly that cue is learned about. Recently, however, several studies used the eye tracking method to study the effect of blocking treatment on overt attention more directly (e.g., Kruschke et al., 2005; Beesley and Le Pelley, 2011; Eippert et al., 2012; also see Wills et al., 2007). Results indicated that subjects spent less time gazing at blocked cues and that dwell time for blocked cues decreased over training blocks. The latter observation suggests that the subjects learn to not attend to the blocked cue during training.

An attentional process related to attentional regulation, attentional bias, might play a role in cue competition as well. Biases in processing threat or reward related information (Bradley et al., 2004; Bar-Haim et al., 2007; Bishop, 2008) might for example have important effects on cue competition, but, to the best of our knowledge, this has not been directly investigated. Still, it is reasonable to hypothesize that a cue that signals a biologically relevant event would take up a disproportionately large amount of attention relative to an innocuous stimulus. Based on this rationale, blocking can be explained by assuming that pairing the blocking cue with an important event in the first phase creates an attentional bias to this cue and, consequently, the blocked cue might come to receive relatively less attention.

### WORKING MEMORY

Working memory is a construct that cognitive psychologists use to account for why informational processing is limited and refers to a process that accounts for temporary storage and manipulation of information necessary to carry out complex tasks (e.g., Baddeley, 1986).

An established strategy to assess whether a task depends on use of working memory is to overburden working memory with a second task and to examine the effect of doing so on performance to the task of interest. Relying on this strategy, De Houwer and Beckers (2003) observed that a working memory load decreased forward blocking performance. In a first experiment they overburdened working memory during the acquisition phase only, whereas they overburdened working memory during both the acquisition and the test phase in a second experiment. The pattern of results was alike in both experiments, a high working memory load reduced blocking relative to the control group, but comparisons with the control group only reached significance in the second experiment. However, Waldmann and Walker (2005) also reported a significant decrease in blocking following administration of a secondary task load during only the acquisition phase. In a more recent study, Liu and Luhmann (2013) pinpointed that such cognitive load during acquisition has its effect in the early portions of phase 2 AX+ training. Taken together, these studies point to the important role that working memory has in modulating cue competition effects. More precisely, they suggest that blocking (at least sometimes) reflects effortful processing, something we will return to in the subsection on higher-order reasoning.

### HIGHER-ORDER REASONING

Inferential learning theory (e.g., De Houwer et al., 2005; also see, e.g., Waldmann, 2000) states that conditional reasoning is involved in cue competition. One might indeed argue that the blocking procedure presents the subject with a reasoning problem: The subject has to determine whether or not the blocked cue is (causally) related to the outcome in order to predict whether the outcome is likely to occur in the presence of the blocked cue alone. This suggests that the blocking effect reflects the organism’s reasoning that the blocked cue is an unlikely cause of the outcome. As scientists, we are of course all familiar with the notion that correlation does not mean causation and the blocking stimulus indeed screens off the relation between the blocked stimulus and the outcome. In the inferential learning theory framework, blocking is, more exactly, hypothesized to be an instance of a modus tollens argument: If a conditional statement (“if p then q”) is assumed to be true, and the consequent does not hold (not-q), then the negation of the antecedent (not-p) should be inferred. Subjects in a blocking procedure are assumed to entertain the assumption that a combination of two causes of the outcome should result in an outcome of greater magnitude (i.e., the additivity conditional). Accordingly, participants can infer that the blocked cue is no cause of the outcome following blocking training, because if the blocked cue were an independent cause of the outcome, then the magnitude of the outcome should be greater during compound training than during elemental training. Below are the arguments for the involvement of inferential reasoning in cue competition.

As said, application of the modus tollens argument in the blocking procedures depends on the assumption that a combination of two causes should result in an outcome of greater magnitude. Several experiments indeed demonstrated that providing subjects with information that either confirms or goes against this additivity conditional respectively increases or reduces the blocking effect (e.g., Lovibond et al., 2003; Livesey and Boakes, 2004; Beckers et al., 2005). Among the same lines, forward blocking should be obtained more easily if the outcome presented during A+ training is non-maximal, because only in such case the causal non-efficacy of the blocked cue X can be validly inferred. If cue A and the AX compound result in an outcome with an intensity that corresponds to the maximal possible intensity, the causal non-efficacy of X cannot be validly deduced, because of a ceiling effect. Consistent with this analysis, De Houwer et al. (2002; also see Beckers et al., 2006) observed stronger blocking in a submaximal than in a maximal condition.

The forward blocking effect has moreover proved to be sensitive to the scenarioa in which the contingency training is embedded. In a predictive learning procedure, participants are asked to rate a predictive relation between a cue and an outcome, whereas in a causal learning procedure, participants are asked to rate the causal relation between a cue and an outcome. De Houwer et al. (2002; Pineño et al., 2005a) found blocking in a causal learning procedure, but not in a predictive learning procedure. Similarly, Waldmann and Holyoak (1992), Waldmann (2000) showed that blocking is obtained more readily if cues A and X are described as causes of the outcome than when cues A and X are described as effects of the outcome. Arguably, the categorization of cues as causes serves as input for a higher-order reasoning system and facilitates blocking by means of activation of the previously described conditionals such as the assumption that causes tend to have additive effects (e.g., De Houwer, 2009). It should however be added that these findings have not always been consistent; several studies found no effect of scenario (e.g., Shanks and Lopez, 1996; Cobos et al., 2002).

Reasoning is presumably an effortful process: Several influential reasoning theories invoke working memory capacity when explaining reasoning performance (e.g., Baddeley and Hitch, 1974; Johnson-Laird and Byrne, 1991; Rips, 1994). Research indeed demonstrates that the amount of errors in standard conditional reasoning tasks increases when working memory is overloaded (e.g., Toms et al., 1993; De Neys et al., 2005). As previously discussed, working memory load decreases forward blocking performance, as would be predicted by general theories of conditional reasoning (e.g., Johnson-Laird and Byrne, 1991; Rips, 1994). Strictly speaking, these studies only suggest that an effortful process is involved in blocking, but there is no logical reason why conditional reasoning would be the only possible candidate effortful process. Interestingly, however, Vandorpe et al. (2005) presented more direct evidence suggesting that working memory can interfere with the making of a valid inference: Secondary task difficulty modulated the number of subjects that were able to verbally report a valid blocking inference in their study.

### MEMORY

Memory processes play an important role in associative learning in general (e.g., Bouton and Moody, 2004) and, by extension, a variety of memory processes (including memory encoding, memory retrieval, and memory rehearsal) also play a crucial role in cue competition phenomena.

#### Memory encoding

Several influential AFMs suggest that the presence of A on AX+ trials results in the formation of a weaker association or memory trace for stimulus X (e.g., Rescorla and Wagner, 1972; Mackintosh, 1975; Pearce and Hall, 1980). According to these theories, a memory-encoding deficit lies at the basis of cue competition.

#### Memory retrieval

Association formation models emphasize memory retrieval as a crucial factor in cue competition as well. Comparator theory (Miller and Schachtman, 1985; Miller and Matzel, 1988) states that at the time of testing (a) the strength of the association between the tested cue and the outcome is retrieved and mentally compared with (b) the strength of the association between comparator cues and the outcome. For example, in a blocking procedure, in which A+ training is followed by AX+ training, A is X’s comparator cue due to its presence during training of X. As a result of the separate A+ training, the strength of the association between A and the outcome will outweigh the strength of the association between X and the outcome during testing, yielding reduced responding to X and, hence, blocking. So, the strength of responding to stimulus X is determined by comparing the strength of the directly and indirectly (i.c., via A) retrieved X-outcome association. Configural theories (Pearce, 1987, 1994), in their turn, suggest that on AX+ trials an association or memory trace is formed between the AX configuration and the outcome. X at test is considered to be a new configuration, which was never presented with the outcome. However, the conditioned response will generalize from AX to X and the strength of the response will depend on the perceived similarity between AX and X. Cue competition is then the result from a submaximal retrieval of the outcome-memory by X.

Inferential learning theory suggests that subjects need to retrieve episodic memories of A+ trials during either AX+ training or testing in order to make a valid inference about the non-efficacy of cue X (e.g., De Houwer, 2009). Of note, subjects do not need to remember the AX+ trials, because the blocking effect can already be attained during actual AX+ training. In line with this argument, a study of Vandorpe et al. (2007) showed that forward blocking does come to depend on memory for AX+ if realization of the blocking effect during actual AX+ training is interfered with.

More evidence for the role of memory retrieval processes in cue competition comes from a recent study. Boddez et al. (2011) investigated the effect of extinguishing a blocking cue on responding to a blocked cue (i.e., A+ and AX+ training followed by A– training). The results indicated that extinguishing A increased conditioned responding to X. Crucially, this increase was context dependent: Increased responding to X was limited to the context in which extinction of A took place (for related findings in animal subjects see Gunther et al., 1998; Blaisdell et al., 1999). Because memory accessibility is known to be context dependent (contexts can either facilitate or disfacilitate retrieval of specific memories; Bouton, 2002), these findings suggest that the blocking effect depends on memory retrieval of the blocking cue as an effective cause or predictor of the outcome.

#### Memory rehearsal

Another memory process affecting cue competition is rehearsal. Meeter and Murre (2004) distinguish between two forms of rehearsal. A first mechanism is a passive or an indirectly triggered form of rehearsal, more precisely the activation of memory traces evoked by related cues. A second mechanism is an active or, what they call, a “conscious” form of rehearsal: goal-directed, effortful and repeated retrieval of memory associations.

The passive form of rehearsal is closely related to the associative concept of within compound associations. Van Hamme and Wasserman (1994) have revised the traditional Rescorla–Wagner model and Dickinson and Burke (1996) have revised Wagner’s (1981) SOP model so that these models incorporate a within compound association mechanism, which allows these models to explain learning about absent cues. Let us illustrate this idea by means of backward blocking. The Dickinson and Burke (1996) model assumes that, during AX+ training, a within compound association forms between cue A and cue X. Indeed, a significant positive correlation between the strength of memory for compounds and backward blocking has been reported (e.g., Melchers et al., 2004) and interfering with the formation of memory for compounds is known to reduce the backward blocking effect (e.g., Dickinson and Burke, 1996; Aitken et al., 2001). The within compound association allows cue A to activate a mental representation of cue X during A+ training, which can subsequently lead to further learning about X. This mechanism is easily reformulated to passive rehearsal (Meeter and Murre, 2004), where the physically presented cue A activates the associated memory representations of cue X due to a process of spreading of activation. Without going into detail about the aforementioned revised models, this within-compound mechanism (or passive rehearsal mechanism) allows these models to account for backward blocking by means of the additional assumption that the associative strength of a cue that is indirectly activated but absent changes in the opposite direction of cues that are present: the associative strength of A will increase during the A+ trials; consequently, the associative strength of the retrieved but absent cue X will decrease.

Rehearsal, either the active or the passive form, can also account for retrospective cue competition and backward blocking if one assumes that the entire former AX+ compound trials are rehearsed during the subsequent A+ trials (e.g., Chapman, 1991; Melchers et al., 2004). Ludvig et al. (2010) presented an extension of the Rescorla–Wagner model in which it is assumed that subjects mentally replay previous trials with consequences similar to actual presentations of those trials. This allows them to explain backward blocking because replaying AX+ trials during A+ training will result in overexpectation and hence a decrement in associative strength of both A and X. For cue A this decrement will however be countered because of the A+ training, whereas this will not be the case for cue X.

In summary, it is clear that a variety of memory processes play an important role in cue competition effects.

### INHIBITION

Cognitive inhibition refers to the suppression or inhibitory regulation of content that is active in working memory (e.g., Harnishfeger, 1995; Nigg, 2000; Kipp, 2005). Accordingly and perhaps not surprisingly, cognitive inhibition has been implicated in reasoning. More precisely, it has been claimed that inhibition of irrelevant thoughts and inappropriate beliefs is central to human reasoning (e.g., De Neys and Van Gelder, 2009). Such inhibition is possibly also involved in the higher-order reasoning processes that can lead to cue competition effects: Thinking about a cue undergoing blocking training might bring to mind the outcome through memory processes, but a reasoning process could afterward still lead to the conclusion that the blocked cue is actually not (causally) related to the outcome. That is, a logical reasoning process might inhibit and override what people intuitively think of (e.g., De Neys and Van Gelder, 2009). Dual-process theories indeed assume that the so-called rule-based system can inhibit and overrule representations activated by the so-called associative system (e.g., Sloman, 1996). Interestingly, also in the comparator theory framework (Miller and Schachtman, 1985; Miller and Matzel, 1988), the cue does activate the representation of the outcome during testing. As a matter of fact, running of the comparator process presupposes activation of the outcome representation by the cue. It is only after the activation of the outcome representation that the comparator process can determine whether or not the activation of the association will result in responding. This is similar to the interplay between higher-order reasoning and inhibition: Presentation of a cue that underwent cue competition training can bring to mind the outcome through associative memory, but reasoning processes can afterward still lead to the conclusion that the cue is actually not (causally) related to the outcome, which in turn would lead to low responding to the tested cue and, hence, a behavioral display of cue competition.

Response inhibition is different from cognitive inhibition: It refers to the suppression of a prepotent response in favor of performing a subdominant response (e.g., Harnishfeger, 1995; Nigg, 2000; Kipp, 2005). Such inhibition can be described as the mechanism that results in the containment of prepotent behavioral responses when such responses are inappropriate or incorrect (e.g., Burle et al., 2004; Ridderinkhof et al., 2004). The discussion of this process is left for the general discussion, because convincing arguments or evidence for its involvement in cue competition are currently still lacking.

## SECTION 2: A COGNITIVE STAGE FRAMEWORK FOR CUE COMPETITION

The present section of this paper describes an integrative framework for understanding cue competition effects. The cognitive processes discussed in the first section of the paper serve as building blocks to engineer this framework.

The framework contends that behavioral display of cue competition is cognitively construed following three cognitive stages that include (1) the encoding stage, (2) the retention stage, and (3) the performance stage. Arguably, these three cognitive stages are essential in understanding cue competition effects. We contend that later behavioral display of cue competition can be achieved, undone or affected in all three stages. The output of each stage serves as input for the next stage. This implies a cascade mechanism: If later behavioral display of cue competition is not achieved at an earlier stage (e.g., encoding), then it may still occur at a later stage (e.g., retention or performance). Similarly, if later behavioral display of cue competition is successfully achieved at an earlier stage (e.g., encoding), it may still be undone at a later stage (e.g., retention or performance). Different cognitive mechanisms are involved in the framework, some of which operate only in certain stages, while others operate in multiple stages. As will become clear, the framework incorporates assumptions and mechanisms of several established learning models.

**FIGURE 1:**
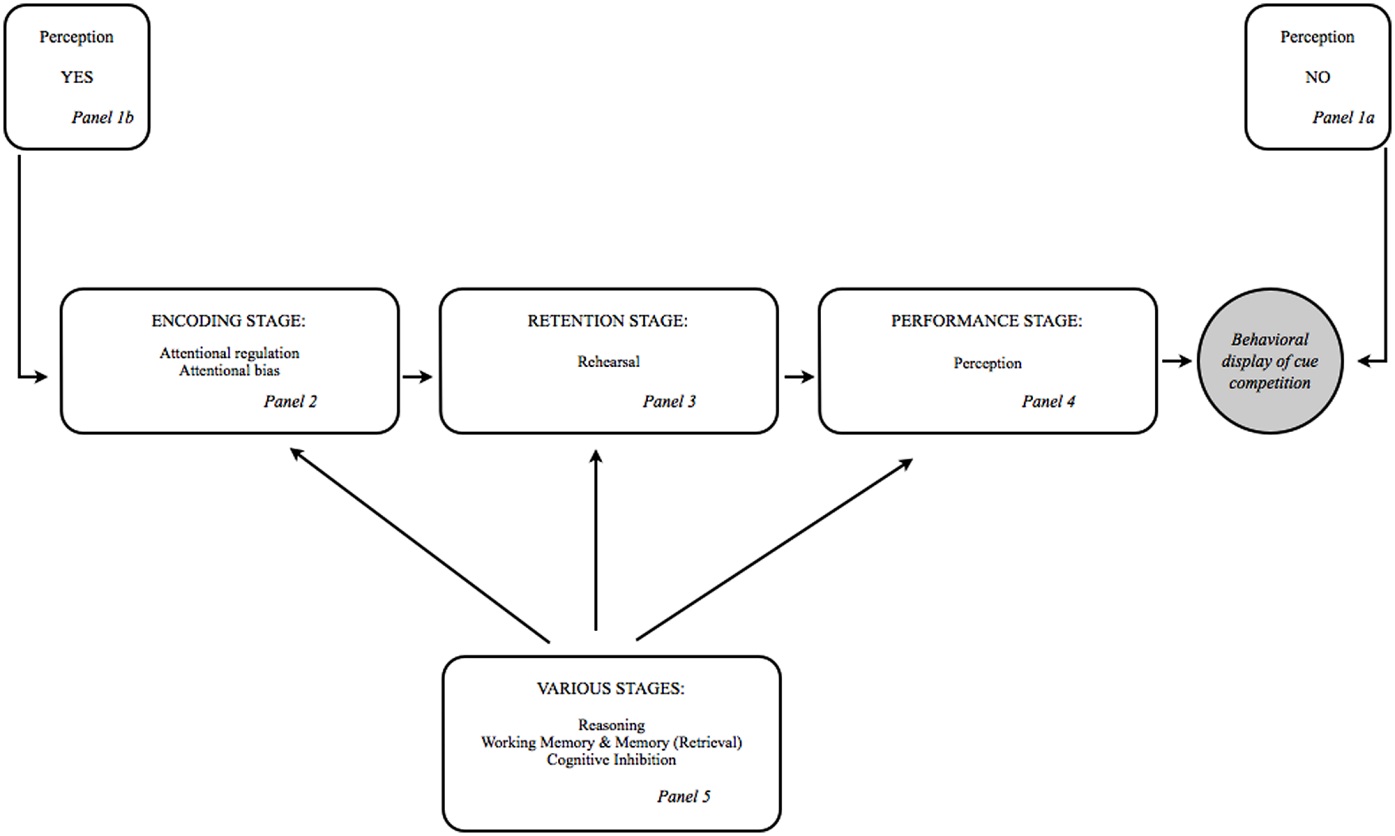
**A cognitive stage framework for understanding cue competition effects in associative learning.** See section one of the present paper for a discussion of the processes and their relevance in cue competition procedures and see section two of the present paper for details concerning the dynamics of the stage framework.

**Figure 1** summarizes the framework, with both the processes that are involved in only the encoding stage, the retention stage or the performance stage, and the processes that are involved in multiple stages. Needless to say, such framework is always incomplete, and like any theory always incorrect, in that many aspects of the real world are inevitably ignored or idealized. Still, we believe that the present stage framework may support a comprehensive understanding of cue competition effects.

### ENCODING STAGE

In this subsection, we focus on the processes operating during the encoding phase of the learning episode.

Panels 1a,b in **Figure 1** represent the gateway function of perception in our framework. Panel 1a in **Figure 1** shows that a failure to perceptually process a cue during a learning episode is the earliest potential source of cue competition effects in the stage framework: The subject must obviously detect the stimulus during the learning episode in order to come to respond in its presence (see Pearce and Bouton, 2001, for a similar argument).

If the cue is perceptually detected during training, other processes come into play, as indicated by panel 1b of **Figure 1**. More precisely, attentional regulation processes and attentional bias are next up in the line of cognitive processes that may affect or result in cue competition effects (see Panel 2 of **Figure 1**). Attentional regulation allows to direct attention to information that is relevant to task demands (e.g., a non-redundant blocking cue) and allows to turn attention away from superfluous information (e.g., a redundant blocked cue). Such attentional processes might lead to cue competition effects under the assumption that the amount of learning and therefore responding to a cue is modulated by the amount of attention paid to such stimulus (e.g., Mackintosh, 1975). Under the same assumption, attentional bias might lead to cue competition effects as well: In a blocking procedure, the previously trained cue A might take up a disproportionally large amount of attention during AX+ training. This appears to be a distinct possibility in conditioning procedures with a biologically significant outcome, as threat and reward related attentional biases have been reliably demonstrated in a variety of experimental paradigms (Bradley et al., 2004; Bar-Haim et al., 2007).

The present framework asserts that a complete failure to process a cue (either because of lack of perceptual detection or as a result of attentional mechanisms) would produce an irreversible acquisition deficit, such that retrospective revaluation of that cue (e.g., recovery from blocking by extinguishing the blocking cue; e.g., Blaisdell et al., 1999) should not be observed. Accordingly, this is also the single exception to our assumption that behavioral display of cue competition that is achieved at an earlier stage may still be affected or undone at a later stage. This occurs because stimuli that are not processed at an early stage do not gain access to memory (e.g., Kastner and Ungerleider, 2000; Mitchell et al., 2006) and retrospective revaluation does require the presence of memory associations between the cue at hand and either the outcome or other cues that were present during training.

### RETENTION STAGE

In this part, we turn to the processes at play in the timeframe between encoding and performance. A crucial assumption of the stage framework we propose is that successful cue competition can be affected in a chain of subsequent information processing stages. If later behavioral display of cue competition is not realized through processes at play in the encoding stage, it can still be attained in the retention or performance stage. Similarly, successfully achieved cue competition can also be affected or undone in later stages (except in the case where cue competition results from a complete failure to process the cue of interest during the encoding phase).

Panel 3 in **Figure 1** indicates the presumed importance of rehearsal processes during the retention stage. Rehearsal, either the active or the passive form (Meeter and Murre, 2004), can for example lead to retrospective cue competition if events that involve a competing cue are rehearsed (e.g., backward blocking; see Ludvig et al., 2010) or to recovery from cue competition if the cue of interest is passively rehearsed in the absence of the outcome (e.g., release from overshadowing due to extinction of the competing stimulus; Dickinson and Burke, 1996). This illustrates that the relation between a blocked cue and an outcome can change in the time between learning and testing, and this without additional training with the blocked cue itself.

### PERFORMANCE STAGE

In this subsection, we will discuss processes that modulate responding to a cue that underwent cue competition training. Before discussing these processes, we will describe how existing theories account for performance of learned behavior.

Provided that cue competition has been successfully achieved in the encoding or retention stage, the only challenge left is to demonstrate so during testing. We will first discuss how the traditional AFMs (e.g., Rescorla and Wagner, 1972; Mackintosh, 1975; Pearce and Hall, 1980) explain low responding to a cue that underwent cue competition training. Most AFMs assume that cue competition training hinders development of a strong link between cue and outcome. This allows these models to provide a straightforward explanation for low responding to a cue that underwent cue competition training: A weak link between cue and outcome implies weak responding. It should however be noted that the link formation account of how responding is generated is somewhat problematic, because it has been demonstrated that there is no one-on-one mapping between associative strength and performance (Rescorla, 2001, 2006).

Still building on the case where cue competition has been successfully achieved in the encoding or storage stage, inferential learning theory holds a rather different view on retrieval and response generation. Mitchell et al. (2009) theorized that learning itself is the consequence of non-automatic inferential reasoning, whereas retrieval of such learning can result from automatic memory retrieval. So, if subjects in for example a blocking procedure, are able to make a valid inference about the blocked cue before testing, they should be able to retrieve that acquired knowledge automatically. An experiment in which a dual task load is applied during only the test phase of a blocking procedure might put this hypothesis to the test, but has -to the best of our knowledge- not yet been carried out. However, Morís et al. (2014) recently demonstrated blocking using a recognition priming test (a test which depends on automatic retrieval processes), providing some evidence for this possibility. It should be noted that response generation is as challenging to inferential learning theories as it is to AFMs: Responses are assumed to be the behavioral expression of beliefs entertained by the subject, but how this translation is done is poorly understood (Baeyens et al., 2009; Mitchell et al., 2009).

Up until now we have considered the case in which cue competition had been successfully achieved in either the encoding or the storage phase, which implies that response generation was the only challenge left. The framework we propose, however, asserts that cue competition can also be affected at the performance stage itself (See panel 4 in **Figure 1**).

While perceiving a cue is obviously necessary for learning about it, recognition of the similarity or difference between a cue at the time of testing and learning is also a critical perceptual phenomenon. That is, cue competition may not only involve cases of failing to perceive a cue during training, but failure to perceive it at test as well (due to a change in percept; Pearce, 1987, 1994). As a sidenote, it is worth mentioning that learning theorists have recently speculated about the role of attention during performance as well (e.g., Kruschke, 2001; Craddock and Miller, 2014) and it is hoped that future studies will deliver supporting evidence that can result in updating of the present framework.

### MULTIPLE STAGE PROCESSES

In this subsection, we focus on the processes that operate in multiple stages. Panel 5 of **Figure 1** gives an overview of the processes that cannot be tied down to a single stage: higher-order reasoning, working memory, episodic memory, memory retrieval, and cognitive inhibition.

Inferential learning theorists have argued that information about different events is stored in memory in a non-competitive manner and that this stored information can then be retrieved and used in a flexible manner by a higher-order reasoning system (Beckers et al., 2006; De Houwer et al., 2007). From this perspective, inferential learning theory advocates a performance-focused view of cue competition, very much like for example comparator theory (Miller and Schachtman, 1985; Miller and Matzel, 1988). But, proponents of the inferential reasoning account add that reasoning processes may also be operating during the initial learning episode or in the timeframe between initial learning and performance (e.g., De Houwer and Beckers, 2003; De Houwer et al., 2005). Thus, under the appropriate boundary conditions, reasoning processes may affect cue competition during all three stages. Particularly interesting is the previously discussed study of De Houwer and Beckers (2003), in which a heightened response to a blocked cue was observed when a dual task load was administered during both training and testing (Experiment 2), whereas this increase was not significant when a dual task was administered in the training phase only (Experiment 1). De Houwer and Beckers (2003, p. 355) offer an interpretation for the difference between their Experiment 1 and Experiment 2 that fits nicely with the stage framework we propose: “It is therefore possible that participants in the difficult secondary task condition of Experiment 1 compensated for the lack of opportunity for deductive reasoning during the learning phase by engaging in more deductive reasoning during the test phase. Such a compensatory strategy was less likely in Experiment 2 because a secondary task was also present during the test phase.” They indeed argue that if later behavioral display of cue competition is not achieved during the encoding stage, it can still be achieved during the performance stage, a central tenet of the framework proposed here.

Effortful cognitive processes rely on working memory capacity. This turns working memory to a multiple stage process, because the present stage framework suggests that effortful processing may be at play during all stages. Let us illustrate. Attentional regulation, at play during the encoding stage, depends on working memory (e.g., Kane et al., 2001; Engle, 2002; Schmidt et al., 2002). A similar argument concerning the supportive or enabling role of working memory can be made with respect to active rehearsal (e.g., Hasher and Zacks, 1979) at play during the retention stage, and higher-order reasoning (e.g., Baddeley and Hitch, 1974; Johnson-Laird and Byrne, 1991; Rips, 1994), at play during the performance stage, amongst others.

As discussed, episodic memory and memory retrieval are also at play during all three stages. Even more so, we should also recognize that subjects do not enter the encoding stage as a blank slate. Subjects get into the encoding stage with a set of earlier experiences and prior knowledge that might have important effects on cue competition, especially in ecologically valid situations. For example, suppose a person, eating potato chips and listening to the theme song of his favorite television show, notices warplanes flying overhead, followed by an air bombing attack. In such case, episodic memory of prior knowledge concerning warplanes might turn these warplanes to a strong competitor.

Finally, cognitive inhibition impacts several other cognitive processes, which turns it to a multiple stage process in the current framework. Let us illustrate with some examples. Attentional regulation, at play in the encoding stage, has been described as an instantiation of an inhibitory process, as it keeps attention focused on the task at hand (e.g., Kane et al., 2001). Cognitive inhibition has also been implicated in reasoning. More precisely, it has been claimed that inhibition of irrelevant thoughts and inappropriate beliefs is central to human reasoning in order to prevent interference (e.g., Markovits and Barrouillet, 2002; De Neys et al., 2005). As discussed, presentation of a cue that underwent cue competition training may for example bring to mind the outcome through memory processes, but interplay between inhibitory and higher-order reasoning processes might still lead to the conclusion that the cue is actually not (causally) related to the outcome, which would lead to low responding to the tested cue.

## GENERAL DISCUSSION

The present paper is built on two pillars. First, we argued that cue competition is no unitary phenomenon, but that different processes are at play in a cue competition procedure. Existing models typically invoke just one or two processes, which may lead to an oversimplified view and to ignoring interplay. Second, we organized processes known or assumed to be involved in cue competition effects in a stage framework. We now turn to the evaluation of this framework.

### PARSIMONY

One may argue that it is unnecessary to invoke a myriad of processes to explain cue competition effects, when parsimonious AFMs (e.g., Rescorla and Wagner, 1972; Mackintosh, 1975; Pearce and Hall, 1980; Miller and Schachtman, 1985; Miller and Matzel, 1988) can explain such effects as well. Although the class of AFMs might indeed do a good job at accounting for cue competition effects, many of the findings discussed in this paper are beyond the scope of individual AFMs. AFMs that characterize cue competition effects as reflecting an irreversible acquisition deficit (e.g., Rescorla and Wagner, 1972; Mackintosh, 1975; Pearce and Hall, 1980) can for example not explain that what is retrieved from long term memory determines whether or not cue competition will be displayed during testing (e.g., Boddez et al., 2011). AFMs that characterize cue competition as a performance phenomenon (Miller and Schachtman, 1985; Miller and Matzel, 1988), to give another example, can in their turn not explain the attentional shifts observed during initial cue competition training (e.g., Beesley and Le Pelley, 2011). Similarly, there are demonstrations of blocking that are out of scope of inferential learning theory. Lovibond et al. (2003), for example, observed a small forward blocking effect in their non-additive group, so under circumstances when it was not logical to infer non-efficacy of the blocked cue. So although the present stage framework might not be very parsimonious, we believe that this is warranted because a more parsimonious theory or framework should only be preferred above a less parsimonious one if both have the same explanatory power. A possible way to bring the sometimes intense debates about cue competition (acquisition versus performance accounts; AFMs versus inferential accounts) to an end might be to let go of the assumption that cue competition is an unitary phenomenon and to consider the possibility that different processes can underlie cue competition effects.

### BOUNDARY CONDITIONS AND EMPIRICAL VALIDATION

Because we argue that cue competition effects can be caused by different processes, it is highly important to identify the boundary conditions that determine which process will be responsible for a cue competition effect in a particular situation. This is an important challenge for future research. The framework we propose already provides some hints about these boundary conditions. A testable prediction derived from the current framework is that recovery from cue competition is possible, except when it is caused by a processing failure during encoding. One could set up an experiment where one induces cue competition due to a processing failure in one condition but not in a second condition. One way to induce such failure is to use many different trials, each with a large number of filler stimuli (Vandorpe and De Houwer, 2006; McLaren et al., 2014). Another possibility would be to use the dot-probe task to manipulate attention away (MacLeod et al., 2002) from the target cue X. In the second condition, one could use a standard blocking task in which such processing failure is rather unlikely (Blaisdell, 2003). The current framework holds that cue competition due to a processing failure is resistant to revaluation. So, a manipulation such as extinguishing the blocking cue A (Boddez et al., 2011) would not produce an increase in responding to the target cue X in the first condition. However, in the second condition, cue competition would remain reversible, because it is hypothesized that cue competition due to other mechanisms than a processing failure can be affected in the other stages of the current framework. Accordingly, recovery from cue competition can be used to inform us about the underlying process in a particular situation. Other boundary conditions include that the possibility of attaining cue competition during the retention stage depends on the presence of a memory trace between the cue of interest and either the outcome or other cues that were present during training (see Mitchell et al., 2006). Finally, achieving cue competition at the performance stage is only possible if the predictive or causal status of competing cues is retrieved from long-term memory (Boddez et al., 2011).

One might moreover predict that individual differences in the processes discussed in this paper will covary with the amount of cue competition. Various measures exist which can tap into these individual difference variables. For example, questionnaires such as the attentional control scale (Derryberry and Reed, 2002) may be used to predict the strength of cue competition effects in humans. Another instance of “individual differences” is the difference between non-human and human animals. It is sometimes argued that animals do not possess the complex cognitive processes of humans. Although it is possible that cue competition in animals is due to only a subset of the processes discussed in the framework, we do not see a reason to make such argument *a priori*. Indeed, there is some evidence to support the idea that rats are able to reason about cues and outcomes (Beckers et al., 2006; Blaisdell et al., 2006) and, in addition, the framework might inspire further studies. To assess the role of working memory, for example, one might develop a task to compare cue competition under single- and dual-task load conditions in animals.

Future research should also focus on the interaction and the temporal dynamics of the different processes. We tried to provide insight in the dynamics of the different processes by means of the stage framework (see **Figure 1**) and by describing the way in which the different processes are interdependent (see Multiple Stage Processes). However, this is only a first step and there is a clear need for empirical data addressing the time course of blocking-related processes (for a great example see Liu and Luhmann, 2013).

In addition, future research could focus on response inhibition, a process left out of the present formulation of the stage framework because its involvement in cue competition is currently still too speculative. Response inhibition refers to the suppression of a prepotent response in favor of performing a subdominant response (e.g., Harnishfeger, 1995; Nigg, 2000; Kipp, 2005) and is an essential element of the response selection mechanisms that contribute to adaptive behavior and accurate performance (e.g., Roberts et al., 1998). Under the assumption that responding to a cue is the prepotent response and withholding responding is the subdominant response, response inhibition might very well be involved in cue competition effects. Preliminary evidence for this assumption comes from an animal study showing that subjects tend to respond rather than not respond whenever a source of uncertainty is thrown into the course of events. It was indeed found in an animal study that subjects showed high responding to a cue that underwent cue competition training when testing took place in an ambiguous context (Gunther et al., 1998). In associative terms, our suggestion implies that a cue that undergoes cue competition treatment is endowed with both excitatory and inhibitory properties. Research has shown that inhibitory regulation is attenuated by shifts in the (physical or temporal) context and by presenting the outcome by itself before testing (for a review, see Bouton, 2002). Accordingly, researchers often rely on demonstrations of such attenuation when looking for evidence for inhibition (e.g., Miguez et al., 2014). Most interestingly, animal studies have demonstrated that inserting a retention interval between training and testing (i.e., a spontaneous recovery procedure; Kraemer et al., 1988; Cole et al., 1997; Pineño et al., 2005b) and presenting the outcome by itself (i.e., a reinstatement procedure; Balaz et al., 1982) strongly decrease cue competition. Such findings are out of scope of current learning models, but fit with the suggestion that response inhibition is involved in the performance stage of cue competition.

### INTERACTION WITH OTHER DISCIPLINES

Association formation models have their own language, which might have isolated them to a certain degree from other domains in psychology where the cognitive revolution has left more permanent marks. Researchers interested in for example attentional control might not make use of cue competition procedures merely because they do not realize that attentional control is largely synonymous to what learning psychologists call learned inattention. Still, cue competition remains among the most intensively investigated effects in learning psychology, which establishes it as a thoroughly explored method for examining several phenomena.

Neuroscientists that use blocking as a tool to gain insight in the neural correlates of prediction error and low-level associative mechanisms (e.g., Steinberg et al., 2013) might be interested to learn that, in fact, multiple higher-order cognitive processes are involved in blocking. Neuroscientists interested in memory plasticity might in their turn benefit from the use of retrospective revaluation procedures in which cue competition effects are altered without any additional training involving the cue of interest (e.g., Corlett et al., 2004; San-Galli et al., 2011; Sharpe and Killcross, 2014).

The framework we propose is also in line with findings from developmental research. Several of the abovementioned functions (e.g., working memory and inferential reasoning) develop during childhood (Greenberg et al., 1977; Byrnes and Overton, 1986, 1988; Cowan, 1997). In late adulthood functions like working memory, associative memory formation and memory retrieval deteriorate (Hartman et al., 2001; Mutter et al., 2006, 2012; Old and Naveh-Benjamin, 2008). Research has shown that cue competition emerges alongside the development of these functions. Levels of cue competition are associated with the development of reasoning abilities and working memory (Simms et al., 2012; McCormack et al., 2013; also see Livesey et al., 2013). Mutter et al. (2012) observed decreased levels of cue competition in older adults, associated with deterioration in associative memory formation and retrieval. Future developmental research can be expanded to other processes described in the framework.

The present framework can also encourage cross talks between fundamental learning research and clinical research. Blocking has been reported to be diminished or even abolished in individuals with schizophrenia (e.g., Jones et al., 1992; Bender et al., 2001), which has sparked the suggestion that blocking may be a useful preclinical laboratory model for studying symptoms associated with schizophrenia (Moran et al., 2008). Among the same lines, Boddez et al. (2012) demonstrated that blocking in aversive conditioning is a valuable tool for gaining insight in the threat appraisal and generalization processes gone awry in pathological anxiety. A deficit in blocking, or a deficit in cue competition at the more general level, indeed results in fear to become disconnected from the most likely causes or predictors of danger. Anxiety and schizophrenia might exert their effects through one or more of the processes described in the present framework. An important challenge for future research is therefore to precisely determine the mechanisms that cause variation in individuals that are at risk for or suffer from pathology.

## CONCLUSION

In sum, we hope that the reader will agree with our conclusion that cue competition effects are driven by a flexible and dynamic system. Arguably, a variety of processes will need to be considered when the story of cue competition is finally written. We also hope that the present paper might inspire to breach some barriers. Numerous cognitive processes have not yet been thoroughly explored in cue competition procedures. Still, our understanding of both these processes and cue competition effects might increase from doing so.

## Conflict of Interest Statement

The authors declare that the research was conducted in the absence of any commercial or financial relationships that could be construed as a potential conflict of interest.
